# Identifying asymptomatic cases during the mass COVID-19 vaccination campaign: insights and implications for policy makers

**DOI:** 10.2217/fvl-2021-0243

**Published:** 2021-12-15

**Authors:** Francesco Chirico, Gabriella Nucera, Olayinka Ilesanmi, Aanuoluwapo Afolabi, Michal Pruc, Lukasz Szarpak

**Affiliations:** ^1^Post-graduate School of Occupational health, Università Cattolica del Sacro Cuore, Rome, Italy; ^2^Health Service Department, Italian State Police, Ministry of the Interior, Milan, Italy; ^3^Department of Emergency, Fatebenefratelli Hospital, ASST Fatabenefratelli & Sacco, Milan, Italy; ^4^Department of Community Medicine, College of Medicine, University of Ibadan, Ibadan, Oyo State, Nigeria; ^5^Department of Community Medicine, University College Hospital, Ibadan, Oyo State, Nigeria; ^6^Polish Society of Disaster Medicine, Warsaw, Poland; ^7^Institute of Outcomes Research, Maria Sklodowska-Curie Medical Academy, Warsaw, Poland; ^8^Maria Sklodowska-Curie Bialystok Oncology Center, Bialystok, Poland

**Keywords:** asymptomatic, coronavirus disease, COVID-19, diagnostic, super spreader, vaccination

Asymptomatic COVID-19 infections have a substantial role in the spread of SARS-CoV-2. Although often overlooked, asymptomatic persons may be contagious and facilitate transmission of SARS-CoV-2 to healthy people. Asymptomatic COVID-19 cases can transmit SARS-CoV-2 to others, although it has been reported that these patients are responsible for fewer secondary infections than symptomatic cases [1]. According to Oran *et al.*, asymptomatic cases account for 40–45% of SARS-CoV-2 infections and can transmit the virus to others for an extended period, probably longer than 14 days [2,3].

In the literature, there is evidence that the high viral load, rather than symptoms, might be the predominant driver of SARS-CoV-2 transmission [4]. Noteworthy is the fact that the viral load of asymptomatic COVID-19 cases is comparable to or higher than that of symptomatic cases [5,6]. Therefore, asymptomatology and high viremia can coexist and thus constitute a major risk factor for the transmission of COVID-19 infection. Asymptomatic people could be; therefore, be described as the main drivers of the ongoing COVID-19 pandemic because they do not manifest symptoms that could raise one’s index of suspicion regarding their COVID-19 status.

Identifying asymptomatic cases as drivers of the COVID-19 pandemic during the mass COVID-19 vaccination campaign is relevant. This may influence the public health decision-making process by governments and policymakers, which should be drawn from evidence-based information. During the ongoing mass vaccination campaign against COVID-19, there is much debate among public health stakeholders about the duration of immunity conferred by the COVID-19 vaccine as well as the development of herd immunity subsequently after vaccination. Available COVID-19 vaccines are incredibly effective in preventing a progression of SARS-CoV-2 infection among people. The recent B.1.617.2 (Delta) variant of SARS-CoV-2 is however a ‘dangerous variant’, and much knowledge is yet to be unraveled about this variant. As events unfold, there exists the likelihood that the Delta variant of SARS-CoV-2 will spread across the population, with resulting dire consequences [7].

The Delta variant of SARS-CoV-2 was identified in India in late 2020 and has subsequently been detected in approximately 60 countries [8]. The Delta variant has a potentially higher rate of transmission compared with other SARS-CoV-2 variants [9]. In July 2021, 469 COVID-19 cases were identified among Massachusetts residents, of which 346 (74%) of these cases had been fully vaccinated. Testing identified the Delta variant in 90% of specimens from 133 patients [10]. Therefore, the US CDC confirmed that fully vaccinated persons could transmit SARS-CoV-2 if infected with the Delta strain despite the >90% protection against hospitalization and death guaranteed by mRNA COVID-19 vaccines (Pfizer-BioNTech and Moderna).

Consequently, the CDC released an updated mask recommendation to ensure that vaccinated persons do not shun public health safety measures. This would help to avert instances in which SARS-CoV-2 gets transmitted to the unvaccinated or immunocompromised [11]. Indeed, it is possible that vaccinated people who get infected may easily remain asymptomatic or present with mild symptoms and thus go unnoticed. Vaccinated people could spread the virus even more insidiously, especially when high viral loads are confirmed. This is because during the COVID-19 mass vaccination campaign, adherence to social distancing by vaccinated people might be disregarded. Therefore, consistent adherence to public health safety measures such as the use of face masks, social distancing measures and practice of hand washing and other hygienic measures is essential. The appropriate use of personal protective equipment (i.e., surgical mask and filtering facepiece 2 in certain high-risk environments, such as crowded places, public transport and indoor workplaces like schools or hospitals, should also be encouraged [10,11]. In addition, occupational health safety practices including health surveillance, screening, testing and contact tracing activities should be promoted [12–14].

During the mass vaccination campaign, identifying the limited number of COVID-19 carriers, asymptomatic or symptomatic, who are responsible for a high proportion of cases (i.e., the ‘super spreaders’) is key for the success of the strategies against the virus [15]. Super-spreaders are people known to be individuals with a high viral load, but not necessarily with COVID-19-related complications. Viral shedding occurs rapidly among the super-spreaders and elimination of their viral load to zero level is delayed [16]. According to Cave, about 10% of super spreaders of SARS-CoV-2 may be responsible for up to 80% of confirmed COVID-19 cases [17]. Exposure to high viral loads may thus result in high-intensity infection, which exposes new cases to high viral loads. Thus, super spreaders generate new super spreaders [18]. Identification of super spreaders should be immediately undertaken through rigorous contact tracing activity, as the number of patients with severe COVID-19 complications declines.

In the next few weeks, new SARS-CoV-2 strains and mutations may emerge. Genomic surveillance to detect new viral strain is therefore necessary. This will help to delay viral shedding as well as reduce the proportion of asymptomatics longer periods [19]. For this reason, testing of symptomatic patients and suspected individuals is vital. Self-testing with antigen tests might be an innovative strategy to promptly identify new COVID-19 cases [20]. SARS-CoV-2 has been shown to be consistently present in saliva. The reverse-transcription PCR of saliva specimens has some advantages over nasal and oropharyngeal swabs, such as cost–effectiveness and avoidance of contact with healthcare workers during specimen collection [21].

Workplace health surveillance should be mounted by local public health agencies in collaboration with occupational physicians to enable prompt testing of individuals. Furthermore, asymptomatic healthcare workers [20] and workers who work on infectious disease wards or COVID-19 isolation centers should maintain high levels of infection prevention and control practices. The general healthcare workers’ population should be encouraged to accept the COVID-19 vaccine. It is yet to be confirmed that available COVID-19 vaccines offer complete immunity against the Delta variant of SARS-CoV-2, thus herd immunity is unlikely until 100% people receive at least two doses of the COVID-19 vaccine [21]. For this reason, vaccinations should be encouraged across the populace and stated as a requirement for high-risk categories such as healthcare workers and fragile persons (aged >50 years, and those affected by comorbidities) who can put the healthcare system under immense pressure.

During the ongoing mass vaccination campaign, there are currently insufficient data for efficacy, safety and effectiveness in children under 16 years of age for Pfizer and under 18 years for the Moderna vaccine. These therefore constitute important ethical concerns. Some cases of myocarditis and pericarditis have also been reported among young males following the administration of the second dose of mRNA vaccines (Pfizer-BioNTech and Moderna). However, the benefits (prevention of severe COVID-19 disease and associated hospitalizations, intensive care units’ admissions and deaths) outweigh the risks (including expected myocarditis after vaccination) in all populations for which vaccination is recommended [22]. Furthermore, published clinical trials do not report any long-term side effect following the administration of these vaccines for emergency use. Another issue concerns people who cannot get vaccinated for medical reasons and will likely remain without protection, especially if herd immunity cannot be attained.

In conclusion, asymptomatic COVID-19 cases could be drivers of the COVID-19 pandemic, thus constituting a public health threat across the globe. Therefore, promoting the use of point-of-care testing and self testing to identify and quarantine asymptomatic people and healthcare workers with a high viral load is needed. While the mass vaccination campaign proceeds, consistent adherence to public health safety measures such as the use of face masks, social distancing measures and practice of hand washing and other hygienic measures is extremely important. Also, the appropriate use of personal protective equipment by healthcare workers and the implementation of health surveillance at workplace should be immediately undertaken. Finally, rigorous contact tracing represents a decisive step in the fight against the COVID-19 pandemic. For this cause, community healthcare workers and community stakeholders should be empowered as focal persons.
